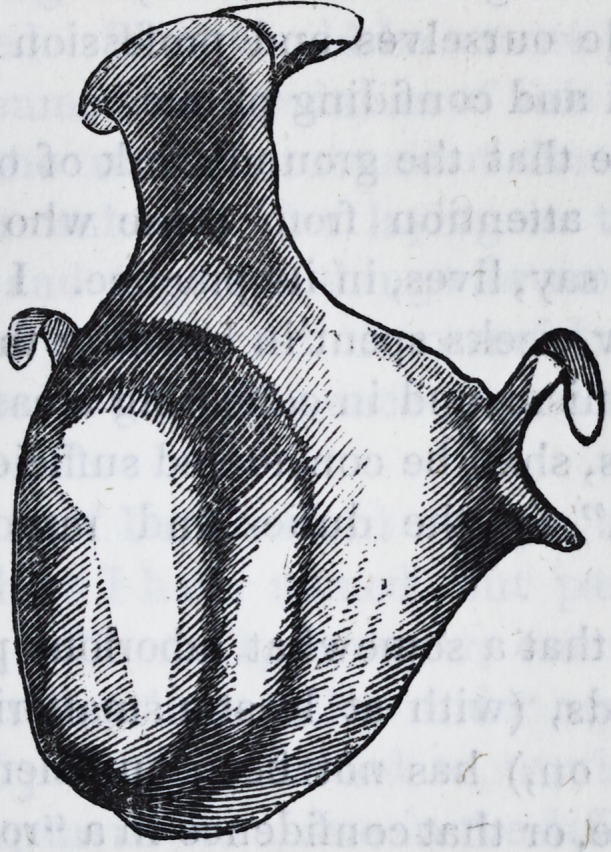# Letter from Dr. A Van Camp

**Published:** 1844-12

**Authors:** 


### ARTICLE X.

### *Letter from* Dr. A. Van Camp.

Nashville, *August 28th,* 1844.

Messrs. Editors.—Should you deem the following worthy of publication in the "American Journal and Library of Dental Science," you will please give it a place in that excellent periodical:

Mr. W., of this city, called on me some weeks since, to have a gold palate repaired, which had been constructed by Dr. B——, of ——, an ingenious and scientific dentist, but which had been subsequently so injured as to render its reparation impracticable. A large portion of the soft palate, the anterior part of the alveolar ridge, and the two upper central incisors were wanting, and it was to supply the loss of these that it had been constructed. The design and execution of the artificial substitute, were such as to reflect the highest credit on the ingenuity and skill of Dr. B., nor will what I am about to say, in the least, detract from the merit of that gentleman; yet, on examining the apparatus, as its restoration to its original adaptation was found tobe impossible, a plan suggested itself to me, by which I thought a new one might be rendered more perfect than this had been. The one constructed by Dr. B., as may be seen by the following cut, which is an exact representation of it, fitted so close to the necks of the teeth, that, even before its adaptation had been destroyed, it induced inflammation and suppuration of the margins of the gums, inflammation and thickening of the dental periosteum, a consequent loosening and soreness of the teeth, and especially of those around which the small hooks had passed:

To obviate this difficulty, I constructed a narrower plate and secured it to the teeth by means of broad clasps. The first was fastened to two of the bicuspides; one on each side of the mouth, the other to the first molares. An outline of this is exhibited in the following cut:

To prevent liquids from passing up into the nose in drinking, although rendered difficult by the motion of the palate, after accurately fitting the plate, I took a separate impression of the opening through it, obtained metallic casts, and stamped up a plate or drum, which extended up through it nearly three-fourths of an inch, that exactly fitted to the orifice of the aperture. This I soldered to the other plate, which, with this addition, presents the following appearance:

This not only prevented fluids from passing into the nose, but it gave greater firmness to the whole apparatus, as well as to the palate and other parts of the mouth. Articulation, too, was rendered more distinct by it, and deglutition more perfect and easy.

## Figures and Tables

**Figure f1:**
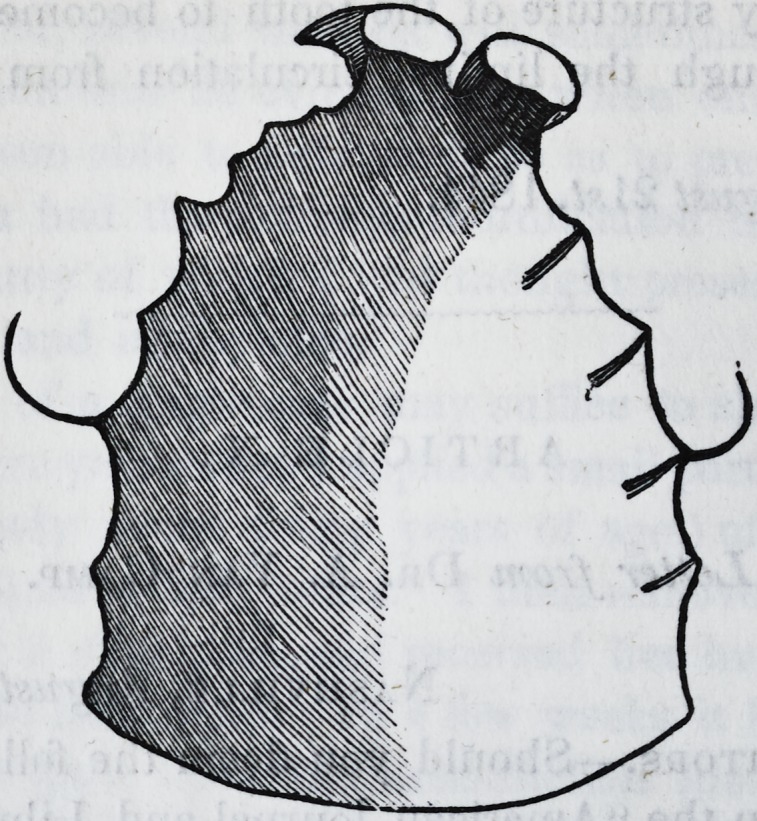


**Figure f2:**
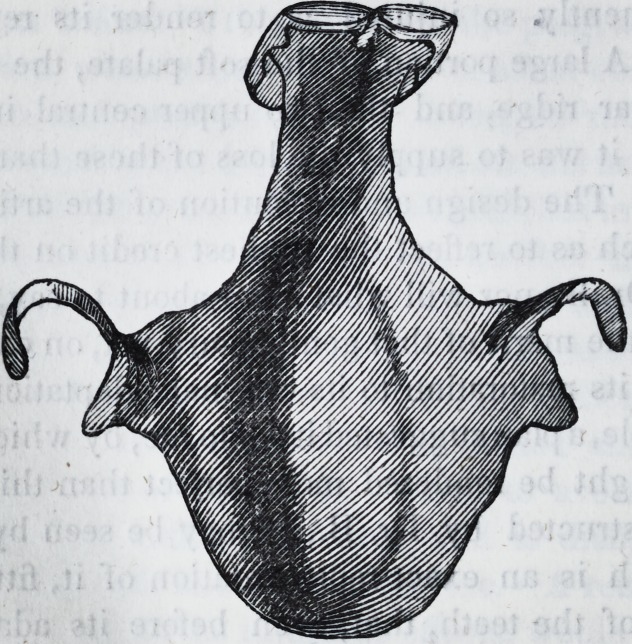


**Figure f3:**